# The pickup of visual information about size and location during approach to an obstacle

**DOI:** 10.1371/journal.pone.0192044

**Published:** 2018-02-05

**Authors:** Gabriel J. Diaz, Melissa S. Parade, Sean L. Barton, Brett R. Fajen

**Affiliations:** 1 The Chester F. Carlson Center for Imaging Science, Rochester Institute of Technology, Rochester, New York, United States of America; 2 Cognitive Science Department, Rensselaer Polytechnic Institute, Troy, NY, United States of America; Purdue University, UNITED STATES

## Abstract

The present study investigated differences in the pickup of information about the size and location of an obstacle in the path of locomotion. The main hypothesis was that information about obstacle location is most useful when it is sampled at a specific time during the approach phase, whereas information about obstacle size can be sampled at any point during the last few steps. Subjects approached and stepped over obstacles in a virtual environment viewed through a head-mounted display. In Experiment 1, a horizontal line on the ground indicating obstacle location was visible throughout the trial while information about obstacle height and depth was available only while the subject was passing through a viewing window located at one of four locations along the subject’s path. Subjects exhibited more cautious behavior when the obstacle did not become visible until they were within one step length, but walking behavior was at most weakly affected in the other viewing window conditions. In Experiment 2, the horizontal line indicating obstacle location was removed, such that no information about the obstacle (size or location) was available outside of the viewing window. Subjects adopted a more cautious strategy compared to Experiment 1 and differences between the viewing window conditions and the full vision control condition were observed across several measures. The differences in walking behavior and performance across the two experiments support the hypothesis that walkers have greater flexibility in when they can sample information about obstacle size compared to location. Such flexibility may impact gaze and locomotor control strategies, especially in more complex environments with multiple objects and obstacles.

## Introduction

To successfully navigate through complex environments in the natural world, humans and other animals must adapt their movements to the conditions that are encountered not only at the present moment but also in the near future. For example, a safety conscious hiker faced with a choice of which of two routes to follow may prefer the one that is initially more difficult if the alternative option eventually leads to a steep slope that lacks secure footing. A hiker who lacks the foresight to account for the dangerous terrain that lies farther ahead will eventually be faced with an unfortunate dilemma: to turn back, thus making an energetically costly corrective adjustment, or to attempt to scale the unsafe slope without injury and at great energetic cost. Similarly, anticipation plays an important role over shorter time-scales as the hiker chooses where to place his or her feet. Sudden and unexpected changes to foot placement are energetically costly, and introduce instability that could lead to falling. Avoiding these sudden adjustments requires the advanced sampling of the visual information necessary for guiding gait in the presence upcoming obstacles in a feedforward manner.

In the present study, we investigated the visual information that walkers use to guide the placement and trajectory of the feet when approaching and stepping over an obstacle. The specific aim was to determine whether there are differences in when visual information about obstacle location and visual information about obstacle size (i.e., height and depth) are needed. We present the results of two experiments designed to test the hypothesis that there is a greater flexibility in the time at which walkers must sample information about obstacle size compared to obstacle location.

### Walkers sample information about the upcoming terrain in advance

Perhaps the most clear-cut example of visual information sampled at one point in time being used at a later time to negotiate obstacles is when guiding the trail foot over the obstacle [1]. Once the lead foot crosses the obstacle, both the trail foot and the obstacle fall outside of the field of view and therefore must be guided based on information that was detected earlier.

Interestingly, walkers use visual information in a feedforward manner well before the obstacle passes out of view. Despite the demands for precision in placement of the feet in front of and behind the obstacle [2–4] and in the elevation of the feet over the obstacle [5], walkers rarely look at the obstacle as they step over it. Instead, they typically glance at the obstacle and the region in front of it two or more steps in advance and then shift their gaze to the regions beyond the obstacle [6, 7]. Although peripheral vision plays a role in negotiating obstacles [8–10], the evidence suggests that information from the lower visual field detected by peripheral vision is also used in a feedforward manner. This was demonstrated using goggles that occlude a portion of the lower visual field (lvf) [4, 11–13]. Subjects can still sample information about the obstacle when they are farther away, but the goggles occlude the obstacle once it is within some range of the subject. In some more recent studies (e.g., [4, 11]), the goggles were fitted with “smart glass” that could be made opaque or transparent with millisecond precision and triggered by force sensors attached to the soles of the subjects’ shoes, which allows for precise control of the timing of lvf occlusion. Using such goggles, Timmis and Buckley [4] demonstrated that occlusion of the lvf upon placement of the trail foot (i.e., the final step) before the obstacle resulted in no changes in toe clearance during obstacle crossing, and no changes in placement of the lead foot on the far side of the obstacle compared to when vision was unobstructed. Similar findings were reported by [14] under conditions in which the entire visual field was occluded at the final step, and by [11] in the context of descending a curb (but see [15] for conflicting findings). This is consistent with the claim that continuous visual sampling is not needed during obstacle crossing—that is, walkers are capable of guiding the feet over the obstacle based on information that was sampled prior to the final step before the obstacle.

Although the act of moving the legs over the obstacle does not appear to require continuous visual sampling, it remains possible that walkers rely on on-line control to guide placement of the feet in front of the obstacle, especially given that variability in foot placement before the obstacle can lead to an increase in the risk of tripping [2, 3, 16]. When lvf occlusion occurs upon or prior to placement of the lead foot (i.e., the penultimate step) before the obstacle, walkers tend to place their feet farther from the front of the obstacle and increase toe clearance compared to when vision is occluded [4, 12]. Likewise, when the lvf is occluded while negotiating an extended stretch of terrain with multiple variations in surface properties, subjects walk slower, take shorter steps, and pitch their heads downward in an attempt to minimize lvf occlusion [17]. These differences likely reflect the adoption of a cautious strategy due to uncertainty about the locations of obstacles relative to the observer, which is important for proper placement of the feet in front of the obstacle.

Although visual occlusion leads to differences in the kinematics of the approach to and step over an obstacle, we contend that it would be premature draw the conclusion that walkers rely on on-line rather than feedforward control to guide placement of the feet on the last step before the obstacle for the following reasons. First, subjects were still capable of successfully crossing the obstacle under such conditions. They simply adopted a more cautious strategy. Second, Patla ([15], Exp 1) manipulated the visibility of an obstacle to be crossed at different points during approach and found that even when the obstacle is made invisible upon placement of the lead foot before the obstacle, there were no differences on key measures except for a small increase in toe clearance (cf. [4, 12, 13]). Thus, the existing evidence is not sufficient to conclude that placement of the trail foot before the obstacle requires continuous sampling. The findings are also compatible with an account that relies on feedforward control.

Of course, there is a limit to how far in advance such information can be sampled. When vision was occluded three steps before reaching the obstacle, subjects cleared the obstacle but with a higher safety margin [14]. Occluding vision five steps in advance dramatically affected the rate of successfully crossing the obstacle [15, 18].

For the purposes of the present study, there are two important points to take away from the prior research. First, although it remains possible that visual information is used in an on-line manner during some phases of obstacle crossing (e.g., possibly to guide placement of the feet before the obstacle), the evidence also indicates that walkers are capable of guiding the lead and trail legs over the obstacle without concurrent visual information. Second, walkers perform best when information about the obstacle is available up until (or shortly before) placement of the trail foot before the obstacle.

### Information about location or dimension?

The previous research summarized above suggests that walkers are most successful in obstacle crossing when they are able to sample information about the obstacle at a particular point during the approach—viz. while initializing and executing the last step before the obstacle. The information that walkers sample at this time appears to be that which specifies the location of the obstacle and is needed to guide placement of the trail foot before the obstacle. When crossing obstacles that have non-negligible height and depth, however, the walker must adapt his or her behavior to the dimensions of the obstacle as well as its location. Hence, in addition to information about obstacle location, walkers must also pick up information about the extent of the obstacle. That is, obstacle crossing requires the detection of both exproprioceptive information (i.e., information about the relation of one’s body to the environment) and exteroceptive information (i.e., information about environmental properties, such as obstacle size) [13, 15]. Whereas walkers are most successful when obstacle location information is sampled at a particular time, it is possible that walkers have greater flexibility in terms of when information about obstacle size can be sampled. This was our specific focus in the present study.

### The present study

#### Aim and rationale

The primary aim of this study was to test the hypothesis that there is flexibility in the time that information about obstacle size can be sampled. Studies of both humans [1] and non-human animals [19, 20] have demonstrated that walkers are able to retain knowledge of obstacle characteristics such as height for an extended period of time. Although memories of obstacle dimensions must be formed during the current approach to be useful [21], this could enable walkers to sample information about obstacle size in advance and use that information to properly elevate the feet during obstacle crossing. Indeed, Patla and Greig [18] found that when vision was occluded five steps in advance of obstacle crossing, subjects elevated their feet to the correct height to clear the obstacle. Although obstacle crossing was unsuccessful on about 50% of trials, the primary cause of failure was an inability to properly place the feet before the obstacle, not an inability to raise the feet to a sufficient height. This suggests that while information about obstacle location must be available when the walker is closer to the obstacle, information about obstacle height can be sampled in advance.

Walkers may also be able to pick up information about obstacle size later in approach. Humans do not place their trailing foot closer or farther from the obstacle across variations in obstacle height [22]. If other gait parameters are invariant across changes in obstacle size leading up to the step over the obstacle, then not knowing the dimensions of the obstacle prior initiation of the crossing step should not affect performance. In a pilot study using a real-world setup similar to the virtual one used in the present study, Parade and Fajen [23] found no differences in step length or walking speed across variations in obstacle height and depth until the step over the obstacle. There were, however, small differences in trail foot placement before the obstacle across variations in obstacle depth, suggesting that additional work is needed to better understand how far in advance walkers need information about the dimensions of the obstacle. Taken together, these findings suggest that walkers may indeed be able to sample information about obstacle size early during the approach (when they are several steps away) or much later (shortly before initiating obstacle crossing) with no significant consequences for their ability to successfully cross obstacles of various sizes.

#### Approach

Our approach was to instruct subjects to step over obstacles in a virtual environment viewed through a head-mounted display (HMD). To test the flexibility in when information may be sampled, we manipulated the availability of visual information about the obstacle’s size. In Experiment 1, the obstacle’s location was always visible by displaying a narrow line at the base of the front edge of the obstacle. In this regard, our approach is similar to that in previous studies in which information about obstacle dimensions was removed while information about obstacle location was made available throughout the approach (e.g., by placing a piece of tape on the floor [21] or adding vertical posts positioned beside the obstacle [24]). However, in the present study, the obstacle itself (and therefore, information about its height and depth) was only visible while the subject was within a narrow visibility window approximately one step length long located roughly four, three, two, or one step(s) away. We refer to these conditions as the VW-4, VW-3, VW-2, and VW-1 conditions, where VW stands for “viewing window”.

If information about obstacle size can be sampled at any point prior to initiation of the step over the obstacle by the lead foot, then there should be no differences in any behavioral or performance measures between any of the first three viewing window conditions and the full vision control condition. There may, however, be differences between the last viewing window condition (VW-1) and the full vision control condition.

## Experiment 1

### Methods

#### Participants

Fifteen undergraduate students from Rensselaer Polytechnic Institute volunteered to participate in the study. Subjects reported that they had normal or corrected-to-normal vision and did not have any visual or motor impairments. The protocol was approved by the Institutional Review Board at Rensselaer Polytechnic Institute and all subjects gave informed consent prior to participation.

#### Apparatus and virtual environment

The experiment was conducted in a 6.5 m × 9 m laboratory. The HMD was an nVis SX111 stereoscopic head mounted display (nVis, Inc., Reston, VA), with a resolution of 1280 pixels × 1024 pixels (nVis, Inc., Reston, VA) per eye and a diagonal field of view of 111 degrees. Head position and orientation were tracked using an Intersense IS-900 motion tracking system (Intersense, Billerica, MA). Data from the tracking system were used to update the position and orientation of the simulated viewpoint. Movements of the rest of the body were tracked using a 14 camera Vicon motion-capture system running at 120 FPS. Subjects wore tight fitting, stretchable clothing to which 34 retro-reflective markers were affixed in accordance Vicon full-body marker template. Subjects completed the experiment barefoot in order to avoid any irregularities caused by differences in footwear. The four Vicon markers normally used to record head position in the full-body template were instead attached to the HMD. The cables from the HMD and tracking system were bundled together and held by the experimenter, who walked alongside the participant as he or she moved to ensure that the cables did not interfere with the subject’s movement.

**Fig 1:**
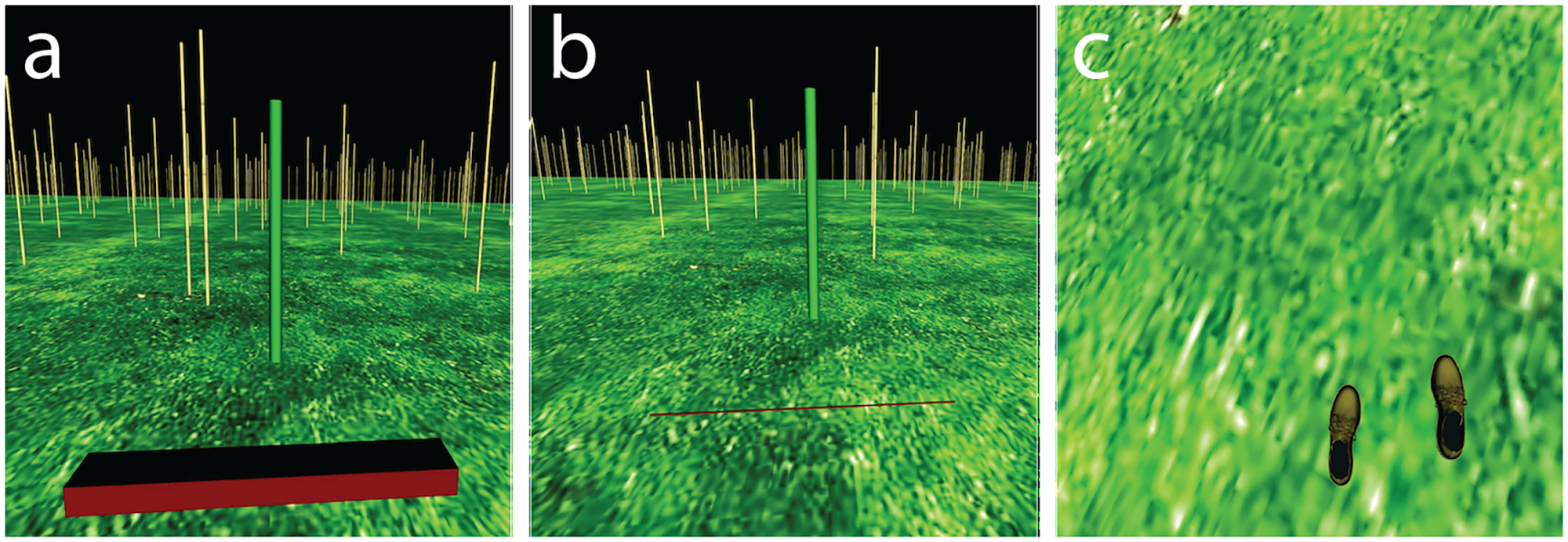
Screenshots of the virtual environment seen through the head-mounted display. (a) The obstacle as seen during full-vision trials or from inside the viewing window. (b) The thin, red line marking the location of the front edge of the obstacle as seen from outside the viewing window in Experiment 1. When the subject was outside of the viewing window in Experiment 2, there was no line indicating the obstacle’s location. (c) The subjects’ virtual feet were visible when the subject looked down.

The virtual environment was created using Vizard Virtual Reality Toolkit (WorldViz LLC, Santa Barbara, CA) running on an Alienware Area-51 PC (Dell, Inc., Round Rock, TX), and consisted of a flat, grass-textured ground plane underneath a black sky (Fig 1A). Bamboo posts were randomly scattered across the ground plane, with the exception of the unmarked 1m-wide path between the start and end positions. The start position on each trial was marked by a translucent box 2 m tall and 0.2 m in width and depth, and the end position was marked by a green cylindrical post positioned 5m from the start box. The end post was always positioned far enough behind the obstacle to allow subjects to take at least one additional step after crossing. When present, the virtual obstacle was a 1m wide red rectangle (see Fig 1A), with height and depth that varied between trials. At certain times during the approach, the obstacle was replaced with a thin red line 1 cm in height and depth and 1 m wide positioned on the ground at the front edge of where the obstacle was located when it was visible (see Fig 1B).

Subjects’ feet were also rendered in the virtual environment and visible through the HMD (see Fig 1C). The dimensions of the subject’s virtual feet were determined by the locations of Vicon markers placed as close as possible to the front of the big toe, the back of the heel, and on the outside of the foot, near to the ankle. The foot’s virtual volume was approximated using a rectangular cuboid that was made coincident with the subject’s real-world foot through a calibration procedure. This procedure involved intentionally aligning the subject’s real-world foot with the x-axis of the capture volume, and calculating the rotation that would subsequently align the foot’s local coordinate system to be in line with the virtual environment’s global coordinate system.

#### Task and procedure

Prior to the beginning of each session, the subject’s leg length was measured as the distance from the greater trochanter of the femur to the floor, and orientation of the virtual foot was aligned to match that of the real-world foot. Subjects were instructed to begin each trial by pressing a button on a hand-held wireless mouse while (1) standing within the visually indicated start box and (2) facing the end post. If these two conditions were met at the time of the button press, a virtual obstacle or line appeared along the subject’s walking path. At the same time, an auditory go-signal indicated that the subject was to begin walking towards the post at a comfortable speed, without stopping, and while stepping over the virtual obstacle along the way.

On some trials, the obstacle was visible only when the subject’s head was within a spatially defined viewing window. When the subject’s head was outside of the viewing window, the obstacle was invisible and was replaced by a thin line on the ground at the location of the obstacle’s front edge. Collisions between the virtual feet and the obstacle were continuously monitored. If any part of either foot collided with any part of the obstacle, an auditory “thud” was played through the speakers.

#### Design

The height, depth, and location of the obstacle were manipulated as independent variables. The values of all three variables were specified in units of leg length (LL) to ensure that the kinematic demands of obstacle crossing would be similar across subjects whose body dimensions varied.

Each experimental session included 126 trials, 46 of which were full vision trials and 80 of which were viewing window trials. Full vision trials varied in obstacle height (0.1, 0.175, 0.25 LL), depth (0.1, 0.2, 0.3 LL), and distance of the obstacle from the start box (3.2, 3.4, 3.6, 3.8, 4.0 LL). Subjects also performed an additional full vision trial to ensure an even number of trials, which was necessary for us to run even and odd numbered trials in opposite directions in the laboratory. On this additional trial, the obstacle was placed at 3.6 LL and had a height and depth of 0.1 LL.

On viewing window trials, information about the obstacle’s dimensions was only available when the subject’s head was inside one of four spatially defined windows. These windows spanned increments of 0.7 leg lengths from the obstacle (i.e. 0-0.7 LL, 0.7-1.4 LL, 1.4-2.1 LL, and 2.1-2.8 LL). Obstacle height (0.1, 0.25 LL), depth (0.1, 0.3 LL), and location (3.2, 3.4, 3.6, 3.8, 4.0 LL) also varied across trials. The selected values of height and depth correspond to the extremes of the ranges used on full vision trials. All independent variables were manipulated within-subjects. All trial types were pooled and presented in a different fully randomized order to each subject. The entire experimental session lasted approximately 45 minutes.

Subjects completed two short practice blocks prior to beginning the experiment. The first practice block comprised 20 full vision trials: 2 obstacle heights (0.1, 0.25 LL) × 2 obstacle depths (0.1, 0.3 LL) × 5 obstacle locations (3.2, 3.4, 3.6, 3.8, and 4.0 LL). In the second practice block, subject completed eight trials, two in each viewing window condition, with both obstacle height and depth set to 0.1 LL. Practice trials were intended to provide familiarity with the virtual environment, and were not included in the analysis.

#### Timing of the viewing window

**Fig 2:**
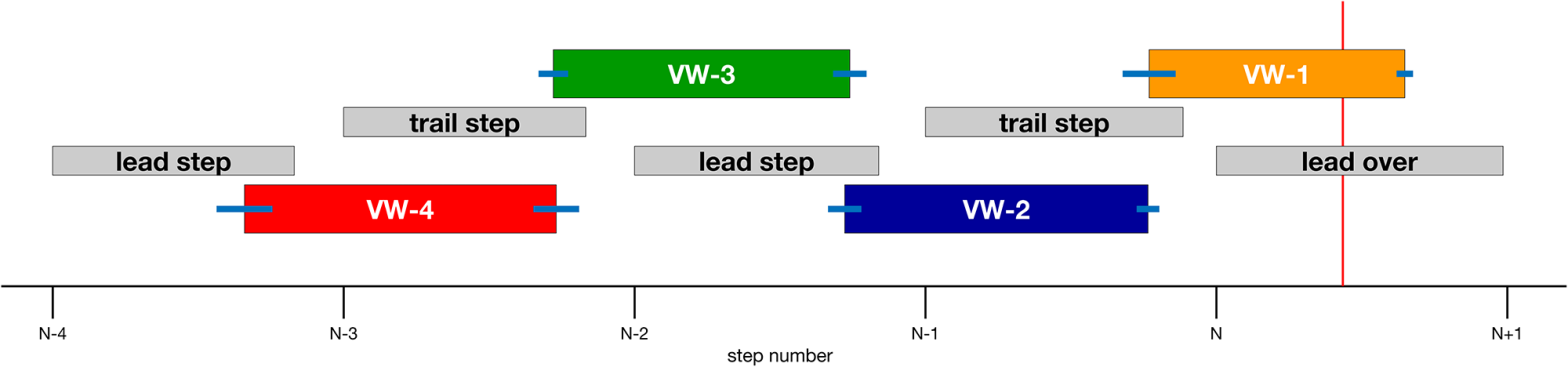
The timing of visibility windows relative to the gait cycle in Experiment 1. Intervals along the x-axis correspond to toe-offs of each step leading up to the step by the lead foot over the obstacle (step N). The left and right sides of each gray box correspond to toe-off and heel strike, respectively, and the length of each box indicates the mean duration of the swing phase scaled to the duration of the step. The left and right sides of each colored box indicate the beginning and end of each viewing window as a proportion of the corresponding step duration, with blue error bars indicating +/- 1 SE. The vertical red line on the right side indicates the mean proportion of the step by the lead foot over the obstacle at which the lead foot first crossed the front edge of the obstacle.

In interpreting the findings below, it is often helpful to know where the subject was in the gait cycle while the obstacle was visible in each viewing window condition. The size of the viewing windows (0.7 LL) was chosen to roughly match average step length during steady state walking [25] so that the obstacle would be visible for approximately one step. Due to variability in step length, the obstacle appeared and disappeared at different points in the gait cycle on each trial. Nevertheless, there was a high degree of consistency in the relationship between the viewing windows and the gait cycle. As shown in Fig 2, in the VW-1 condition (orange box), the obstacle appeared shortly before heel-strike by the trail foot on the last step before the obstacle and remained visible until shortly after mid-swing of the crossing step by the lead foot. In each of the other VW conditions, the obstacle generally appeared toward the end of each step and remained visible until shortly before the end of the next step. Variability in the onset and offset of each viewing window is indicated by the blue horizontal lines at the beginning and end of each colored bar.

#### Analysis

Subsequent to data collection, Vicon Nexus was used to convert marker positions into joint positions. Data were filtered using a seventh-order low-pass Butterworth filter with a cutoff at 4 Hz. Steps were identified using a velocity based kinematic analysis presented in [26] and used in several previous studies [27–29]. The position of toe, heel, and ankle markers on each foot were cast into a body-centered coordinate system by subtraction of the sacral marker, which was placed at the base of the subject’s spine, halfway between the left and right posterior iliac spines. The position of the ankle markers along the axis of locomotion was differentiated to find a velocity signal in which zero-crossings were indicative of the ankle and foot’s velocity relative to the sacral marker, which provided a rough indication of the subject’s COM. These zero crossings were used as indication that the foot’s trajectory had changed direction within the body-centered coordinate system. Transitions from negative to positive velocity are marked as heel strikes, and transitions from positive to negative as toe-offs. The identification of heel strikes had the additional requirement that ankle velocity in the vertical direction was less than 0.5 meters per second. Steps in which the duration was less than 100 ms were removed, as were steps in which foot velocity does not exceed 0.75 m/s in the forward direction. The results of this algorithm were visually inspected for accuracy.

We calculated two measures of obstacle crossing behavior: maximum foot elevation and obstacle clearance. Maximum foot elevation was estimated based on the height of the toe marker at the peak of its trajectory during the crossing step. Obstacle clearance was based on the distance from the toe marker to the top of the obstacle at the moment that the toe marker crossed the front face of the obstacle.

Our approach to analyzing the data relied on mixed-effects regression modeling using the “nlme” package in R. For each dependent measure, we built a set of linear models with various combinations of the three independent variables (i.e., height, depth, viewing window) and in some cases their interactions as predictors. To keep the number of levels of height and depth consistent across all five viewing window conditions, we excluded data from the middle height and middle depth conditions in the full vision condition. Decisions about which models to build and compare were motivated by the theoretical predictions. For most dependent measures, we first compared a model with height as a predictor against the baseline (intercept-only) model. If the height model is superior to the baseline model, this is equivalent to finding that the main effect of height is statistically significant. To test the main hypothesis of the study, we then compared the model with height against a model with both height and viewing window (or a model with height, viewing window, and height × viewing window) as predictors. If behavior (as measured by the dependent variable) is influenced by viewing window or the height × viewing window interaction, the more complex model will be superior to the simpler model. Thus, by comparing the two models, we can determine whether viewing window influenced behavior or if the effect of height varied across viewing window.

Model fit alone is not an adequate metric for determining which of two models is better, since more complex models always better fit the data. Instead, we compared models using the log-likelihood ratio comparison (in the form of a chi-squared significant test) and the change in Akaike Information Criterion (AIC). AIC is a widely used metric of the quality of a model because it captures both the goodness of fit and the complexity of the model. When reporting the difference in AIC (dAIC) between two models, negative values indicate that the model of interest is superior to the comparison (usually less complex) model.

After analyzing the effects of height and viewing window, we repeated the process for depth and viewing window. Thus, these were treated as separate analyses. Technically, it is possible to combine height, depth, and viewing window into a single analysis for each dependent measure. However, such an approach would have required consideration of models with higher-order interaction terms (e.g., height × depth, height × depth × viewing window). This was not necessary because the height × depth interaction was not statistically significant for any of the dependent measures.

### Results and discussion

#### Approach phase

**Number of steps** On average, subjects took 5.59 (SE = 0.13) steps to reach the obstacle in the full vision condition. The mean number of steps was slightly greater in each of the viewing window conditions (M = 5.70 in VW-4, 5.69 in VW-3, 5.75 in VW-2, 5.71 in VW-1) compared to full vision. Indeed, the model with viewing window as a predictor had a significantly lower AIC than the baseline model (*χ*^2^(4) = 13.83, p<.01, dAIC = -5.83). Adding height (*χ*^2^(1) = 1.19, p = .28, dAIC = -.81) or both height and height × viewing window (*χ*^2^(5) = 1.53, p = .91, dAIC = 8.47) did not significantly improve the model. However, the model with depth and viewing window was superior to the model with viewing window (*χ*^2^(1) = 6.02, p<.05, dAIC = -4.02), reflecting a tendency to occasionally take an additional step during approach to deep obstacles (M = 5.73) compared to shallow obstacles (M = 5.64). We attribute this difference to the tendency (which we report in the analysis of foot placement below) to place the feet slightly closer to the obstacle when it is deeper.

**Fig 3:**
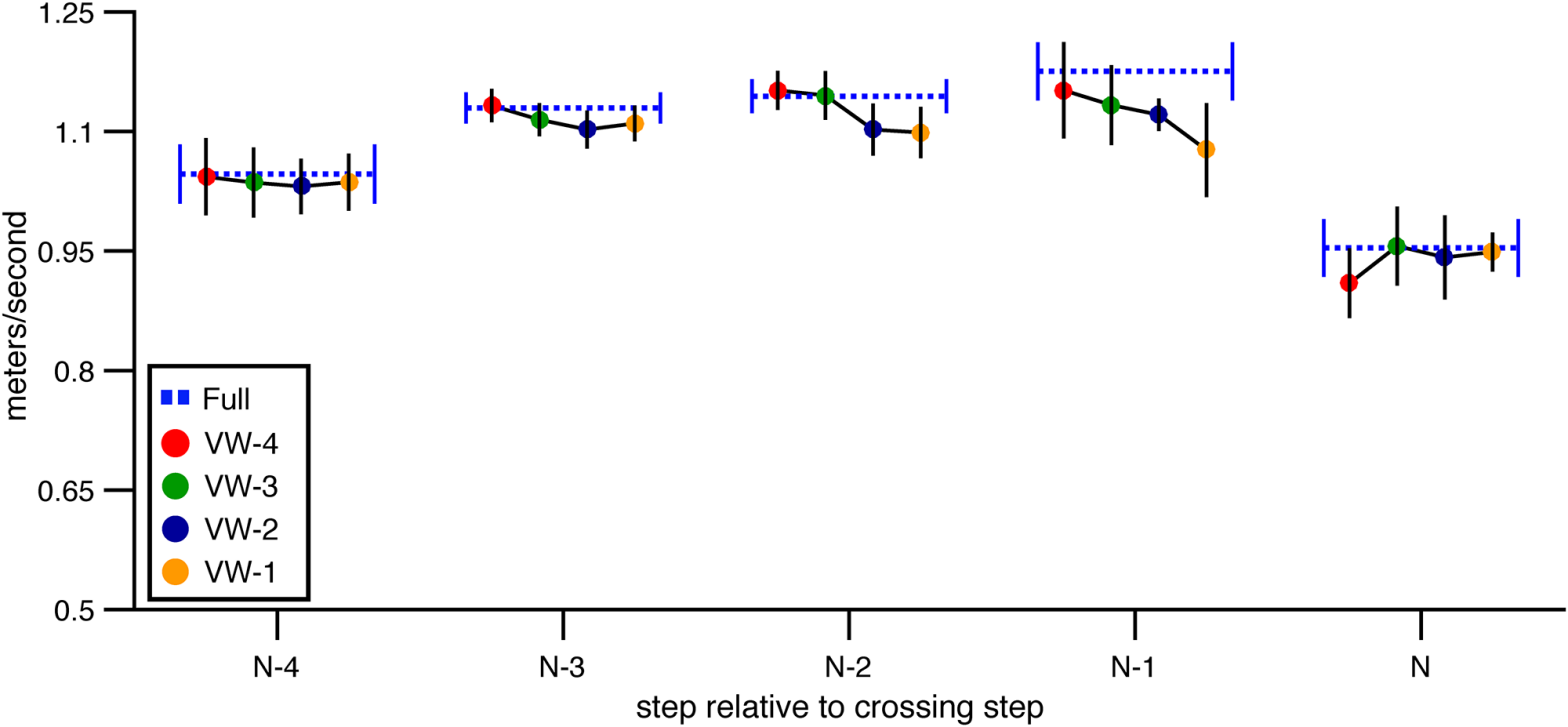
Mean forward velocity of the COM over steps in Experiment 1. Mean forward velocity of the subject’s center of mass as a function of step number in the full vision and viewing window conditions. Error bars reflect 95% confidence intervals.

**Walking speed** In general, subjects gradually accelerated between steps N-4 and N-1, reaching peak walking speed at step N-1 before decelerating on the step over the obstacle (Fig 3). The change in speed over steps was statistically significant, leading to an improvement in the model with step number included as a predictor compared to the baseline model (*χ*^2^(4) = 128.23, p<.01, dAIC = -120.28). Adding height (*χ*^2^(1) = 1.01, p = .31, dAIC = 0.97) or depth (*χ*^2^(1) = 0.22, p = .64, dAIC = 1.78) did not improve the model relative to the step-number model, suggesting that subjects did not walk at different speeds in different obstacle conditions. However, AIC decreased when viewing window and viewing window × step number were included (*χ*^2^(20) = 98.01, p<.01, dAIC = 58.01). Inspection of Fig 3 suggests that forward COM velocity did not vary much across VW conditions and was similar to forward COM velocity in the full vision condition for steps N-4 and N-3. As subjects drew within two steps of the obstacle, a trend began to emerge wherein subjects appeared to walk slower on trials in which the obstacle became visible later. However, the magnitude of the difference in speed was less than 0.1 m/s. Taken together, this analysis suggests that subjects were able to maintain steady forward progress regardless of the viewing window within which information about obstacle height and depth was available. This is consistent with the hypothesis that walkers can sample information about obstacle size in a flexible manner. The small reduction in speed on step N-1 is an exception, and suggests that if information is not made available before the trail foot is placed in front of the obstacle, walkers alter their behavior, perhaps to be more cautious.

#### Foot placement before obstacle

**Fig 4:**
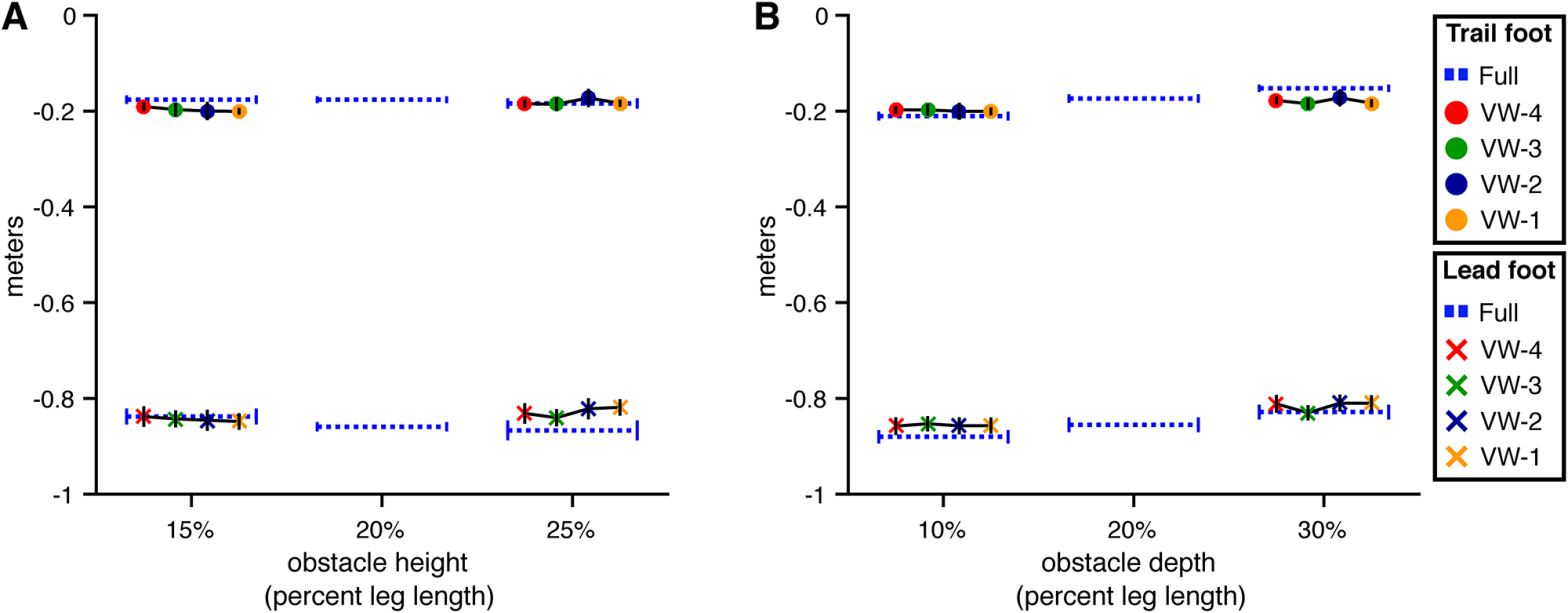
Placement of the lead and trail foot prior to the step over the obstacle in Experiment 1. Each panel presents the distance from the obstacle to the toe marker on the planted lead foot and trail foot prior to the step over the obstacle. Error bars reflect 95% confidence intervals with between-subjects variability removed. (A) Distance by obstacle height and viewing window. (B) Distance by obstacle depth and viewing window.

Fig 4 shows mean foot placement distance in front of the obstacle for both the lead and trail foot as a function of obstacle height (A) and depth (B). The observed distances are similar to those reported in studies of obstacle crossing in the real world, where lead foot placement ranges from 0.9 to 1.0 m [13–15] and trail foot placement ranges from 0.2 to 0.25 m [4, 13–15, 22].

**Obstacle height** For lead foot placement, the model with height as a predictor was not significantly different than the baseline model (*χ*^2^(1) = 0.21, p = .65, dAIC = 1.791) (see Fig 4A). This is consistent with findings from previous studies in which the effect of obstacle height was not significant [18, 22]. The quality of the model did not improve (relative to the baseline model) when viewing window was added as a predictor (*χ*^2^(5) = 5.56, p = .35, dAIC = 4.44), nor did it improve when the model included height, viewing window, and the height × viewing window interaction (*χ*^2^(9) = 15.46, p = .07, dAIC = 2.54). Although the difference between the full model and the baseline model is close to statistically significant, the change in AIC is positive, consistent with a poorer model. Taken together, the analysis reveals no evidence that lead foot placement was influenced by obstacle height, viewing window, or their interaction.

Similarly, there was no evidence that trail foot placement was affected by either variable or their interaction. The model with the lowest AIC was the baseline model. Neither the model with height (*χ*^2^(1) = 1.95, p = .16, dAIC = 0.05), nor the model with height and viewing window (*χ*^2^(5) = 3.40, p = .64, dAIC = 6.60), nor the model with height, viewing window, height × viewing window (*χ*^2^(9) = 10.95, p = .28, dAIC = 7.05) was significantly better.

**Obstacle depth** For lead foot placement, the model with the lowest AIC included only depth as a predictor (see Fig 4B). The depth-only model was significantly better than the baseline model (*χ*^2^(1) = 13.13, p<.01, dAIC = -11.13). Adding viewing window did not significantly improve the model (*χ*^2^(4) = 7.03, p = .13, dAIC = 0.97), nor did adding both viewing window and the depth × viewing window interaction (*χ*^2^(8) = 8.96, p = .35, dAIC = 7.04). Thus, subjects placed their lead foot closer to the obstacle when it was deeper, but lead foot placement was not significantly affected by viewing window or the depth × viewing window interaction.

A similar pattern of results was observed for trail foot placement. The model with the lowest AIC was the one with depth as a predictor. This model was significantly better than the baseline model (*χ*^2^(1) = 13.79, p<.01, dAIC = -11.79), but not significantly different from the model with viewing window (*χ*^2^(4) = 1.48, p = .83, dAIC = 6.52) or the model with viewing window and the depth × viewing window interaction (*χ*^2^(8) = 13.79, p = .09, dAIC = 2.22).

To summarize, positioning of the feet in front of the obstacle was consistent across variations in obstacle height (as in previous studies), but subjects tended to place their feet slightly closer to the obstacle when it was deeper. However, neither lead nor trail foot placement were affected by the viewing window manipulation or its interactions with height or depth. The fact that subjects adapted foot placement to variations in obstacle depth in the VW-4 and VW-3 conditions provides compelling evidence that walkers are able to adapt to variations in obstacle dimensions even when the relevant information is sampled several steps in advance.

#### Obstacle crossing

The next set of analyses focuses on obstacle crossing and is organized by foot (i.e., lead foot first followed by trail foot).

**Fig 5:**
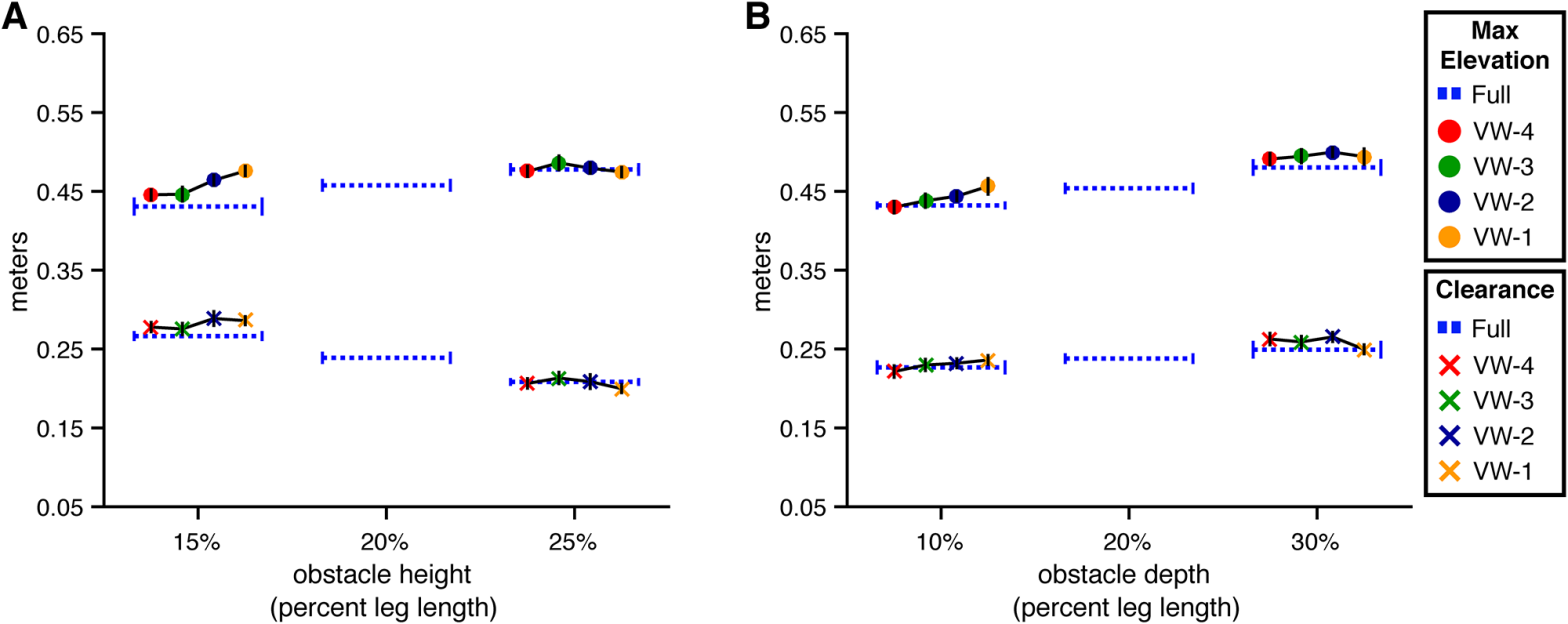
Maximum height and clearance of the lead foot in Experiment 1. Maximum step height and obstacle clearance of the toe marker placed on the lead foot at the time it crossed over the front face of the obstacle. Error bars reflect 95% confidence intervals with between-subjects variability removed. (A) By obstacle height and viewing window. (B) By obstacle depth and viewing window.

**Lead foot elevation and clearance** Let us first consider the effects of obstacle height and viewing window on lead foot obstacle crossing (Fig 5A). The model of lead foot elevation with height as a predictor was significantly better than the baseline model (*χ*^2^(1) = 15.47, p<.01, dAIC = -13.47). This confirms that subjects were able to discriminate obstacles of different heights in the virtual environment and adapt their behavior as they do in the real world. Had subjects not been able to perceive variations in obstacle height (i.e., due to difficulty in VR), they could have successfully performed the task by elevating their feet to the same height (higher than the tallest obstacle). The increase in lead foot elevation with obstacle height suggests otherwise.

However, the model with the lowest AIC was the full model that included height, viewing window, and their interaction. This model was significantly better than the height-only model (*χ*^2^(8) = 20.05, p<.05, dAIC = -4.05), which suggests that the manipulation of obstacle height affected lead foot elevation differently depending on when the obstacle was visible. To unpack the interaction, we conducted four 2 (VW-# vs. Full) × 2 (Short vs. Tall) interaction contrasts using Dunnett’s correction. The interaction was significant (p < .05) in the VW-1 condition but not in any of the other viewing window conditions. This likely reflects adoption of a cautious strategy in the absence of information about obstacle height. If so, this would suggest that walkers are most successful in obstacle crossing when they can sample information about obstacle height prior to placement of the trail foot before the obstacle, which is consistent with the results of the analysis of walking speed.

Although lead foot elevation increased with obstacle height, the change in elevation was less than the change in obstacle height. As such, lead foot clearance decreased with obstacle height (*χ*^2^(1) = 49.82, p<.01, dAIC = -47.82) (see lower half of Fig 5A). This differs from results reported in studies of obstacle crossing in the real world, where foot clearance tends to remain constant across obstacle height. One possible explanation for the difference is that there is greater uncertainty about the position and movement of one’s legs and feet relative to the obstacle in the virtual environment, which could lead to more cautious behavior. Indeed, lead foot clearances in the present study (20-30 cm) were slightly greater than those observed in studies conducted in the real world (12-20 cm [2, 4, 5, 13–15]). Of course, there are limits to how high subjects are able (or willing) to elevate their feet. Hence, maintaining the same clearance for taller obstacles as for shorter obstacles may have required more effort than subjects were willing to expend.

Next, we consider the effects of obstacle depth on lead foot crossing (Fig 5B). The model of lead foot elevation with obstacle depth was significantly better than the baseline model (*χ*^2^(1) = 26.18, p<.01, dAIC = -24.18), which is consistent with the findings of Patla and Rietdyk [5] showing that subjects elevate their feet to a greater height when crossing deeper obstacles. Neither the model that included both depth and viewing window (*χ*^2^(4) = 8.77, p = .06, dAIC = -0.77) nor the full model (*χ*^2^(8) = 15.26, p = .06, dAIC = 0.74) were significantly better than the depth-only model. Thus, unlike the effect of obstacle height, the effect of obstacle depth did not significantly vary across viewing window conditions. The pattern of results was similar (compare Fig 5A and 5B), but in the case of obstacle depth, the interaction did not reach statistical significance.

**Fig 6:**
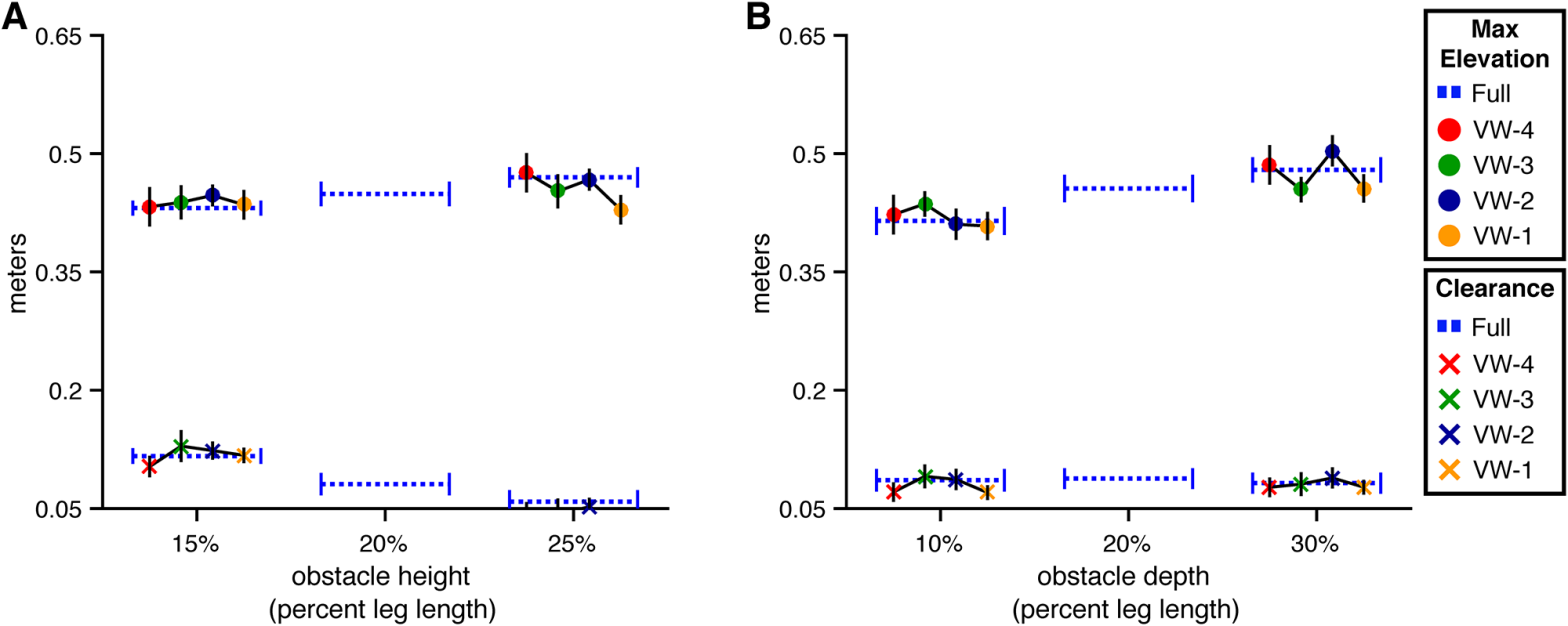
Maximum height and obstacle clearance of the trail foot in Experiment 1. Maximum step height and obstacle clearance of the toe marker placed on the trail foot at the time it crossed over the front face of the obstacle. Error bars reflect 95% confidence intervals with between-subjects variability removed. (A) By obstacle height and viewing window. (B) By obstacle depth and viewing window.

**Trail foot elevation and clearance** Subjects also elevated their trail foot to a greater height when crossing taller obstacles (Fig 6A). This was corroborated by the model comparison analysis, which revealed that the model with height was superior to the baseline model (*χ*^2^(1) = 6.42, p<.05, dAIC = -4.42). As with the lead foot, the increase in trail foot elevation was less than the change in obstacle height, resulting in a significant decrease in trail foot clearance (*χ*^2^(1) = 33.81, p<.01, dAIC = -31.81). Mean trail foot maximum elevation was greater for taller obstacles in each of the viewing conditions except for VW-1, mirroring the pattern of results observed with the lead foot. However, the model of maximum foot elevation with viewing window and height × viewing window was not significantly better than the height model (*χ*^2^(8) = 9.86, p = .27, dAIC = 6.14). In other words, the effect of obstacle height did not vary across viewing window conditions as it did for the lead foot. This is likely due to the greater variability in trail foot elevation.

Trail foot elevation also increased with obstacle depth (*χ*^2^(1) = 12.02, p<.01, dAIC = -10.02) (see Fig 6B). The model of trail foot elevation with depth, viewing window, and depth × viewing window was significantly better than the depth-only model (*χ*^2^(8) = 18.22, p<.05, dAIC = -2.22), suggesting that obstacle depth may have affected trail foot elevation differently across viewing window conditions. Specifically, inspection of Fig 6B suggests that the obstacle depth effect in the VW-3 condition may have differed from the effect in the other conditions. We did not predict this interaction and do not have an explanation for it. Moreover, despite the presence of a significant interaction, none of the follow-up 2 (VW-#) × 2 (Shallow vs Deep) interaction contrasts with Dunnett’s correction were significant (p > .05). Thus, this could be a spurious result.

#### Collision rates

**Fig 7:**
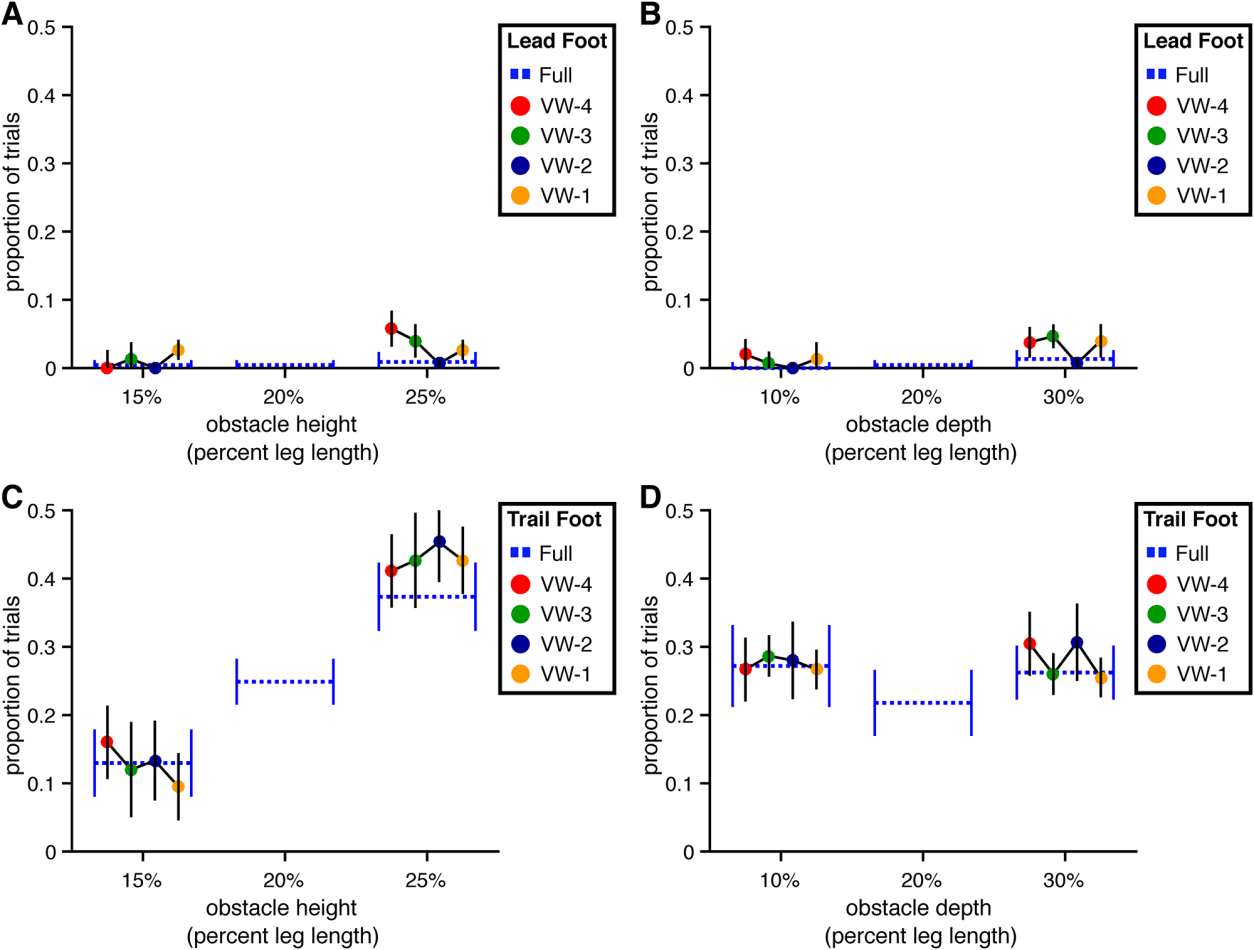
Collision rates for Experiment 1. The percentage of trials with a collision between the obstacle and the lead and trailing foot are presented by obstacle height and depth. Error bars reflect 95% confidence intervals with between-subjects variability removed. (A) Collisions with the lead foot by obstacle height. (B) Collisions with the lead foot by obstacle depth. (C) Collisions with the trailing foot by obstacle height. (D) Collisions with the trailing foot by obstacle depth.

Collision rates for the lead foot were below 2% on average and were not significantly affected by obstacle height (*χ*^2^(1) = 3.60, p = .06, dAIC = -1.60) or viewing window *χ*^2^(4) = 9.23, p = .06, dAIC = -1.24) (see Fig 7A). Subjects did, however, collide with deeper obstacles (2.8%) significantly more often than with shallow obstacles (0.8%) (*χ*^2^(1) = 4.09, p<.05, dAIC = -2.09) (see Fig 7B).

Trail foot collision rate increased with obstacle height (*χ*^2^(1) = 7.72, p<.01, dAIC = -5.72) but was not significantly affected by viewing window (*χ*^2^(4) = 0.71, p = .95, dAIC = 7.29) or depth (*χ*^2^(1) = 0.04, p = .84, dAIC = 1.96) (see Fig 7C and 7D). The rate of collisions involving the trail foot (27.5% overall) is higher than that which has been reported in previous studies conducted in the real world [14, 22]. For the purposes of the present study, however, the absolute collision rate in the full vision condition is less important than the relative collision rate between the visibility window conditions and the full vision condition. Thus, the fact that the trail foot collided with the obstacle more often in the present study than in previous studies does not change the interpretation of results.

#### Summary

Several conclusions can be drawn from these analyses. First, when the obstacle was visible throughout the trial, subjects were able to adapt foot placement and trajectory to variations in the position, height, and depth of the obstacle, much like they do in the real world. The only measures on which performance noticeably differed were foot elevation during obstacle crossing and the frequency of collisions involving the trailing foot.

Second, for most dependent variables, adding viewing window, height × viewing window, and/or depth × viewing window did not improve the model, suggesting that walking behavior was at most weakly affected by the absence of obstacle dimension information during most of the approach. The largest effects were observed when such information was not made available until subjects were within one step length of the obstacle. In this condition (i.e., VW-1), the obstacle first appeared on average shortly before placement of the trailing foot before the obstacle (see Fig 2). The fact that subjects tended to exhibit more cautious behavior (e.g., slowing down, elevating the lead foot to a greater height) in this condition suggests that walkers perform best when they are able to sample information about obstacle height and depth before placement of the trailing foot. However, subjects were still able to cross the obstacle without an increase in collision rate even if such information is made available later, by making subtle changes to enact a more cautious strategy.

Third, there was no evidence that walking behavior or obstacle crossing performance were affected when obstacle dimension information was available early in the approach and then removed (i.e., in the VW-4 and VW-3 conditions). As in full vision, subjects adapted foot placement to obstacle depth but not height, elevated their feet to a greater height for taller and deeper obstacles, and maintained a comparable rate of collisions even in the VW-4 condition. This provides compelling evidence that walkers are able to sample information about obstacle size in advance and use that information several steps later to guide obstacle crossing.

Taken together, the findings of Experiment 1 support the hypothesis that walkers have considerable flexibility in the timing of the sampling of information about obstacle size. Such information can be sampled early when the walker is several steps from the obstacle, which implies an ability to use information that was detected several steps earlier. Alternatively, information about obstacle size can be sampled later, shortly before placement of the trail foot before the obstacle, which reflects the fact that accommodating variations in obstacle size does not require adjustments until shortly before the step over the obstacle is initiated.

## Experiment 2

### Introduction

In Experiment 1, our focus was on information about obstacle size and how such information can be sampled in a brief glance at any point between two and four steps in advance. Our hypothesis, which was motivated by previous research on obstacle crossing, is that such flexibility in the timing of visual sampling is specific to information about obstacle size. That is, information about obstacle location, unlike information about obstacle size, is most useful when it is sampled at a particular point in time during approach—shortly before placement of the trail foot before the obstacle. However, the difference is likely to be a matter of degree; that is, walkers may have some flexibility in terms of when they can sample information about obstacle location, but less flexibility than is afforded for information about obstacle size.

The design of Experiment 1 does not allow us to compare such differences in flexibility because information about obstacle location was continuously available in all five conditions. The results of the previous studies summarized in the introduction provide some insight into the range of distances within which location information is most useful. However, any conclusions would be stronger if they were based on a more direct comparison using the same experimental paradigm. To this end, we conducted a second experiment that was similar to Experiment 1 but with the visibility manipulation applied to both size and location information. In other words, the obstacle was either visible or not. There was no persistent line indicating the position of the front edge of the obstacle. If information about obstacle location is most useful when it is sampled while the walker is within a narrow range of distances from the obstacle, then unlike in Experiment 1, differences between the viewing window conditions and the full vision condition should be widely observed.

### Methods

#### Participants

Nine undergraduate students from Rensselaer Polytechnic Institute volunteered to participate in the study. Subjects reported that they had normal or corrected-to-normal vision and did not have any visual or motor impairments. The protocol was approved by the Institutional Review Board at Rensselaer Polytechnic Institute and all subjects gave informed consent prior to participation.

#### Apparatus, task, procedure, and design

The methods were identical to those of Experiment 1, with the exception that the line at the front edge of the obstacle was removed in Experiment 2. As such, information about obstacle location, height, and depth was only available when the subject’s head was within the viewing window.

#### Timing of the viewing windows

**Fig 8:**
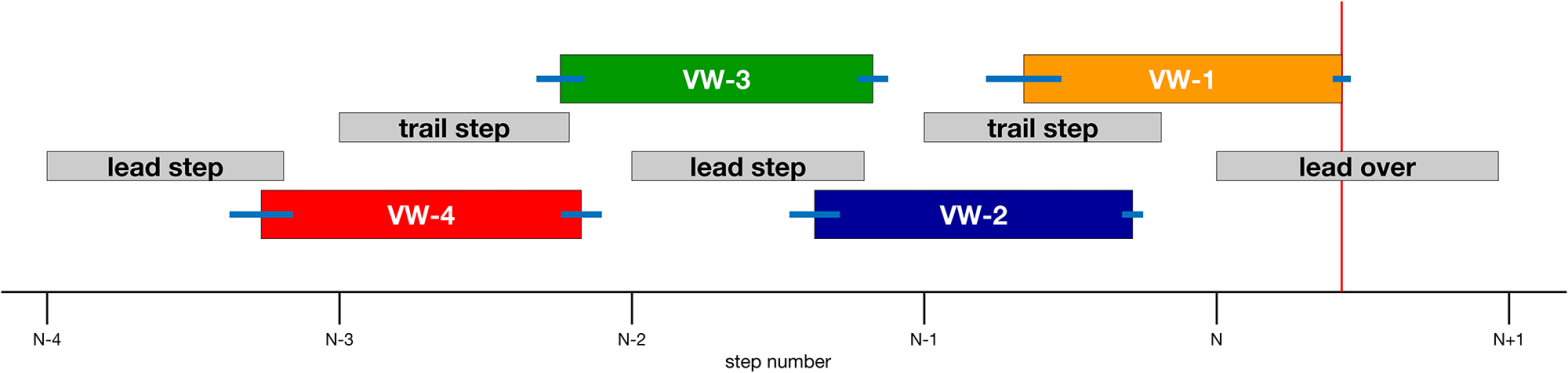
The timing of visibility windows relative to the gait cycle in Experiment 2. Intervals along the x-axis correspond to toe-offs of each step leading up to the step by the lead foot over the obstacle (step N). The left and right sides of each gray box correspond to toe-off and heel strike, respectively, and the length of each box indicates the mean duration of the swing phase scaled to the duration of the step. The left and right sides of each colored box indicate the beginning and end of each viewing window as a proportion of the corresponding step duration, with blue error bars indicating +/- 1 SE. The vertical red line on the right side indicates the mean proportion of the step by the lead foot over the obstacle at which the lead foot first crossed the front edge of the obstacle.

As in Experiment 1, timing of the viewing windows was determined by the position of the subject’s head from the obstacle, defined in units of leg length. The actual time at which the appearance and disappearance of the obstacle were triggered with respect to the events in the gait cycle is presented in Fig 8. Window onset is similar to that in Experiment 1, with the one exception that viewing window VW-1 was triggered and extinguished earlier in the subject’s approach. As we show below, withholding information about obstacle location led subjects to adopt a more cautious strategy across all trials. Oftentimes, subjects took slightly smaller steps and needed to take an extra step before crossing the obstacle. This had the effect of compressing the last viewing window when represented in terms of gait cycle events.

### Results

#### Approach phase

Whereas the viewing window manipulation had little effect on walking behavior during the approach phase in Experiment 1, differences between the VW conditions and the full vision condition were apparent in Experiment 2.

**Fig 9:**
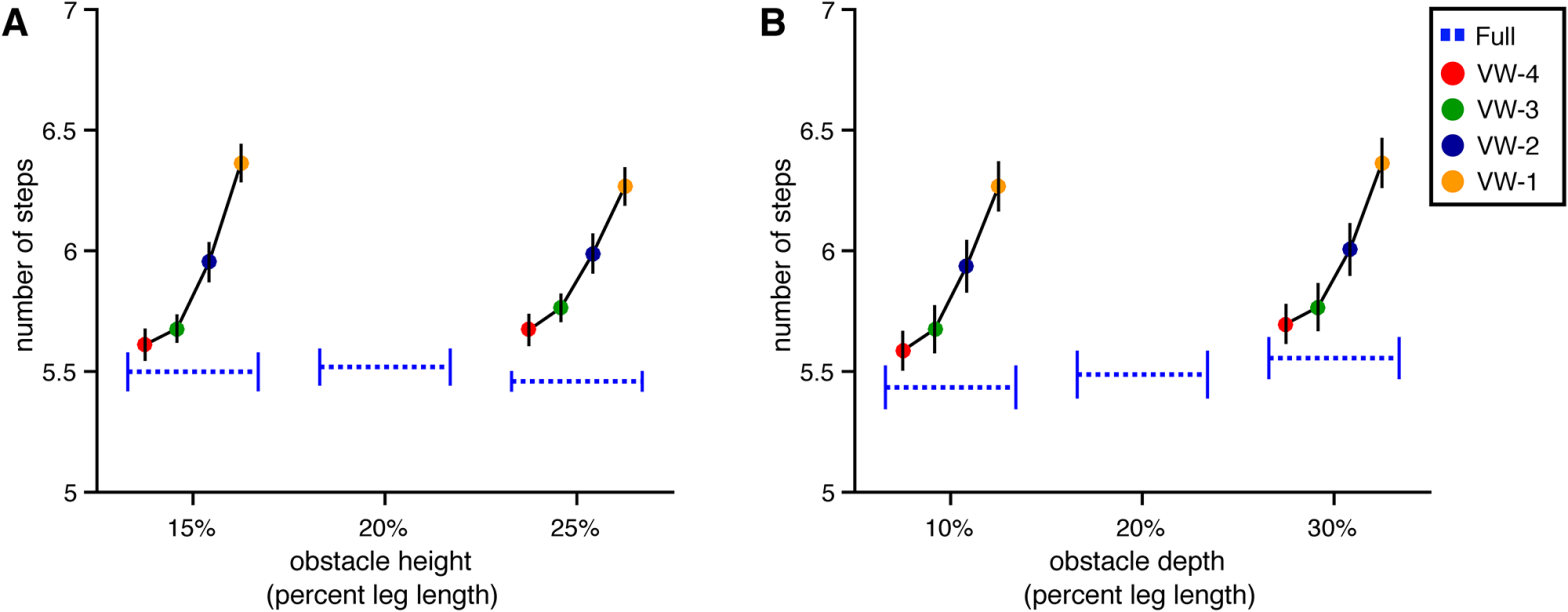
The number of steps prior to obstacle crossing for Experiment 2. Number of steps prior to obstacle crossing for full vision and each viewing window condition. Error bars reflect 95% confidence intervals with between-subjects variability removed. (A) By Height. (B) By depth.

**Number of steps** The number of steps that subjects took before crossing the obstacle was significantly affected by viewing window (*χ*^2^(4) = 95.77, p<.01, dAIC = -87.77). As depicted in Fig 9, subjects took more steps in conditions in which the obstacle appeared later. This is consistent with the analysis of walking speed (reported below), which shows that subjects tended to walk slower in those conditions. Adding height as a predictor did not improve the model (*χ*^2^(1) = 0.05, p = .82, dAIC = 1.95 compared to the model with viewing window), but adding depth did (*χ*^2^(1) = 5.50, p<.05, dAIC = -3.50). As in Experiment 1, the mean number of steps was slightly greater during approaches to deeper obstacles.

**Fig 10:**
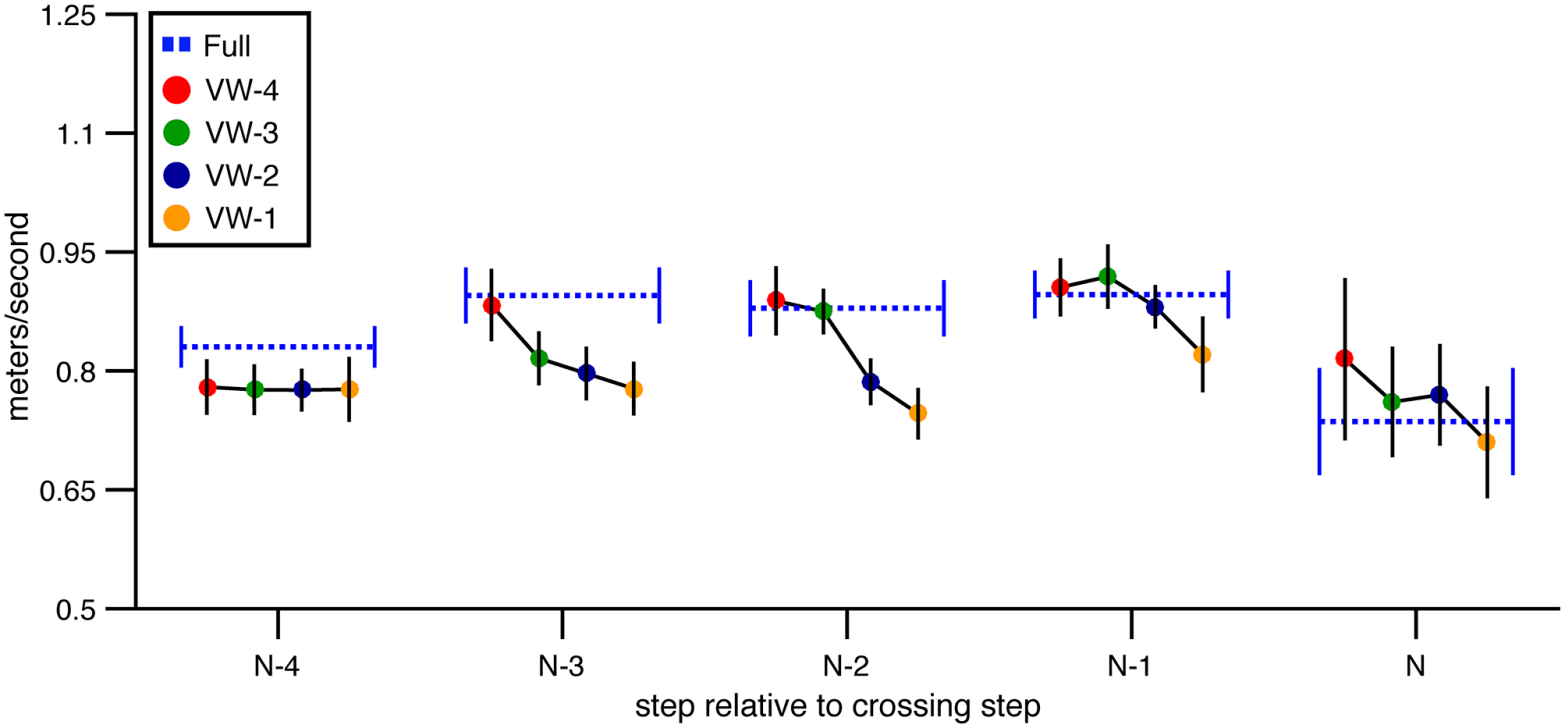
Mean forward velocity of the subject’s center of mass in Experiment 2. Mean forward velocity of the subject’s center of mass is presented for each step of the approach, for full vision and viewing window trials. Error bars reflect 95% confidence intervals with between-subjects variability removed.

**Walking speed** The manipulation of viewing window also affected walking speed (see Fig 10). The model of walking speed with the lowest AIC included step number, viewing window, and step number × viewing window (*χ*^2^(24) = 386.539, p<.001, dAIC = -338.539 compared to the baseline model). Adding either height (*χ*^2^(1) = 0.15, p = .70, dAIC = 1.85) or depth (*χ*^2^(1) = 1.30, p = .25, dAIC = 0.70) did not further improve the model. These analysis suggest that walking speed was consistent across changes in obstacle dimension but that the walking speed profile varied depending on when the obstacle was visible. As depicted in Fig 10, mean walking speed on step N-4 was slower in each of the visibility window conditions compared to the full vision condition. Shortly after the obstacle became visible in each condition, walking speed recovered to full vision levels. This occurred on step N-3, N-2, N-1, and N in the VW-4, VW-3, VW-2, and VW-1 conditions, respectively. Thus, it appears that subjects tended to walk at a reduced speed until the obstacle appeared. Upon obstacle appearance, they accelerated within one step to the same speed as they walked in the full vision condition and maintained that speed even after the obstacle disappeared.

On their own, these findings could be interpreted as evidence that subjects proceeded cautiously due to the absence of information about obstacle size, obstacle location, or both. However, in Experiment 1, which differed from Experiment 2 only in that information about obstacle location was visible throughout each trial, walking speed in the VW conditions was nearly identical to full vision on each step with the exception of step N-1. This suggests that it was the absence of obstacle location information (not size information) that led subjects in Experiment 2 to walk initially at a slower speed.

Interestingly, walking speed was slower overall compared to Experiment 1 even in the full vision condition. We attribute this difference to the adoption of a more cautious strategy in the VW conditions of Experiment 2, and the fact that full vision trials were randomly interleaved with and less frequent than trials in which obstacle visibility was manipulated. Thus, the tendency to walk slower apparently carried over to full vision trials.

#### Foot placement before obstacle

**Fig 11:**
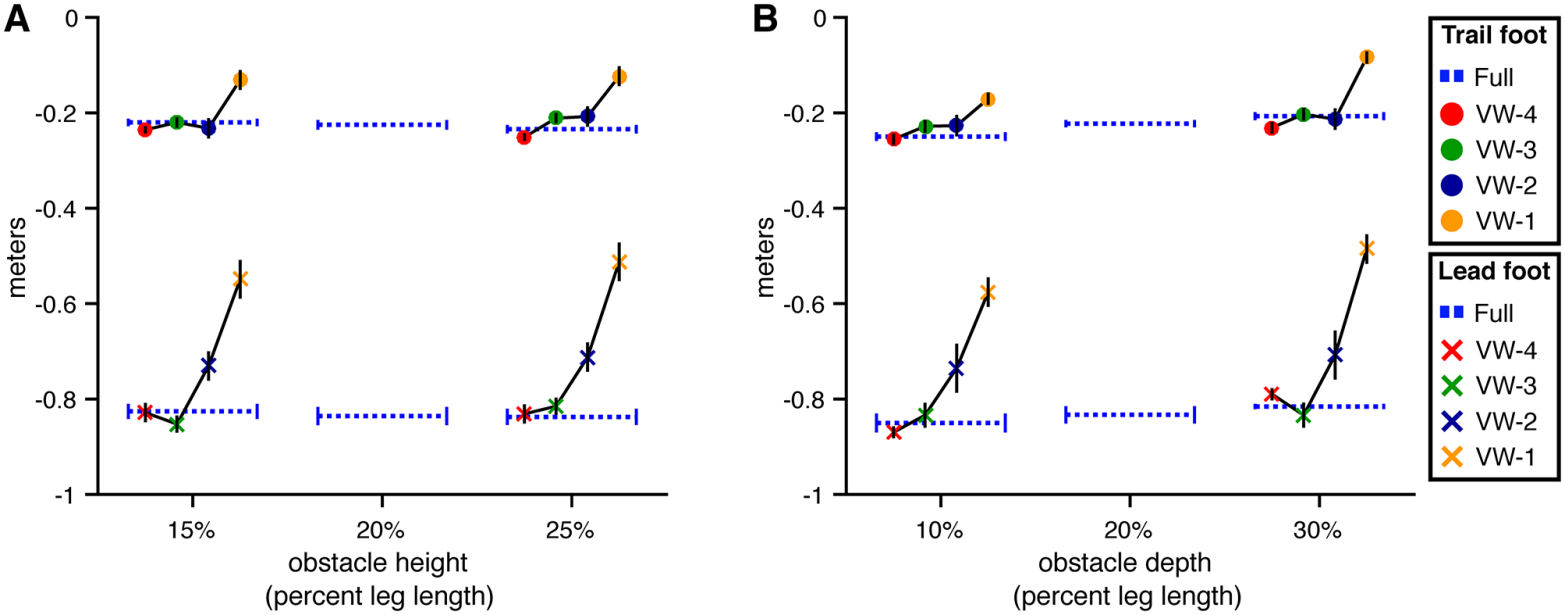
Placement of the lead and trail foot prior to the step over the obstacle in Experiment 2. Each panel presents the distance from the obstacle to the toe marker on the planted lead foot and trail foot prior to the step over the obstacle. Error bars reflect 95% confidence intervals with between-subjects variability removed. (A) Distance by obstacle height and viewing window. (B) Distance by obstacle depth and viewing window.

**Obstacle height** As in Experiment 1, placement of the lead and trail feet before the obstacle on the last step before crossing was consistent across obstacle height (*χ*^2^(1) = 0.26, p = .61 dAIC = 1.74 for the lead foot; *χ*^2^(1) = 0.01, p = .96, dAIC = 2.00 for the trail foot; see Fig 11A). Foot placement was also affected by viewing window. Adding viewing window as a predictor to the height-only model resulted in a significant improvement for both lead foot (*χ*^2^(4) = 116.51, p<.001, dAIC = -108.51) and trail foot (*χ*^2^(4) = 72.70, p<.001, dAIC = -64.70) placement.

Contrasts with Dunnett’s correction between each VW condition and the full vision condition revealed significant differences (p < .05) in the VW-2 and VW-1 conditions for the lead foot and in the VW-1 condition for the trail foot (see Fig 11). Thus, in the VW-4 and VW-3 conditions, when the obstacle was visible several steps in advance and then removed, subjects placed both feet in roughly the same location as they did in the full vision condition. At first glance, this result seems at odds with results of studies showing that when vision was occluded 3-5 steps in advance, subjects were more cautious [4, 11–13]. The difference is likely due to the fact that in our study, the obstacle disappeared but the surrounding environment, including texture on the ground, remained visible. It is possible that subjects used texture elements near the obstacle as landmarks to keep track of the obstacle’s location after it disappeared (see [30] for a similar effect in the context of memory-guided reaching). The presence of a richly textured environment surrounding the obstacle may have also benefited subjects by enhancing stability and postural control [31].

In the VW-2 condition, subjects placed their lead foot significantly closer to the obstacle compared to full vision, but trail foot placement was not significantly different than full vision. These effects were observed for each combination of obstacle height and depth. This makes sense because in the VW-2 condition, the obstacle did not appear on average until shortly before the end of the lead foot’s last step before the obstacle (see Fig 8). Thus, subjects did not have sufficient time to fully adjust placement of the lead foot to the position of the obstacle. However, trail foot placement in VW-2 was very similar to that in full vision, indicating that subjects were able to adapt within about one step.

In the VW-1 condition, both the lead and trail feet were placed significantly closer to the obstacle than in full vision. This also makes sense because in the VW-1 condition, the obstacle did not appear until after placement of the lead foot and shortly before placement of the trail foot.

**Obstacle depth** Lead foot placement was not significantly affected by obstacle depth (*χ*^2^(1) = 2.41, p = .12, dAIC = -0.41) but trail foot placement was significantly closer when the obstacle was deeper (*χ*^2^(1) = 9.64, p<.01, dAIC = -7.64) (see Fig 11B). Adding viewing window as a predictor to the depth-only model resulted in a significant improvement for both lead foot (*χ*^2^(4) = 110.00, p<.001, dAIC = -102.00) and trail foot (*χ*^2^(4) = 69.13, p<.001, dAIC = -61.13) placement.

Contrasts with Dunnett’s correction between each VW condition and the full vision condition revealed significant differences (p<.05) in the VW-2 and VW-1 conditions for the lead foot and in the VW-1 condition for the trail foot. It is noteworthy that subjects placed their trail foot closer to the obstacle when it was deeper in the VW-4 condition, as they did in the full vision condition (see Fig 11B). This provides additional evidence that subjects were able to sample information about obstacle depth when they were several steps from the obstacle and use that information later to adapt foot placement.

The results of the analysis of foot placement before the obstacle are not particularly surprising. Nevertheless, the contrast with the results of the same analysis for Experiment 1 (Fig 4) highlights how this key aspect of successful obstacle crossing is disrupted when information about obstacle location is not available during approach.

#### Obstacle crossing

**Fig 12:**
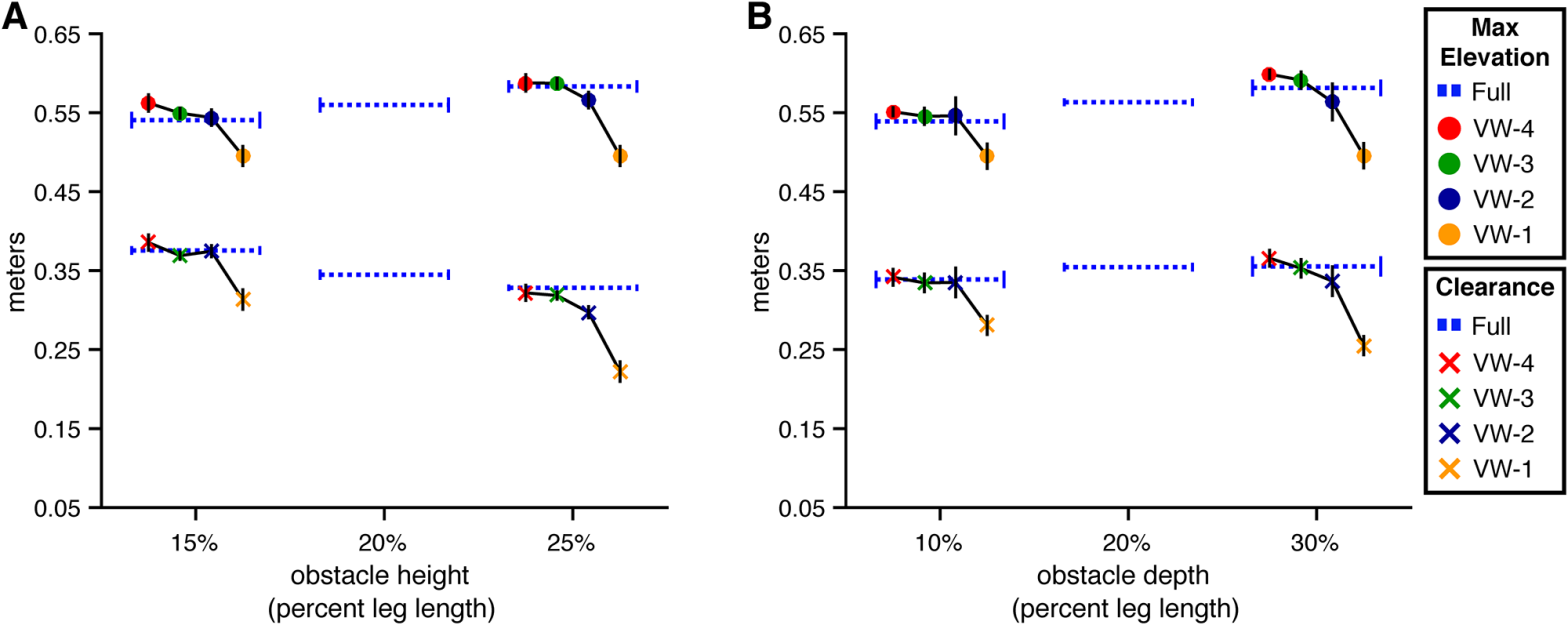
Maximum height and clearance of the leading foot for Experiment 2. Maximum step height (top) and obstacle clearance (bottom) of the toe marker placed on the foot at the time it crossed over the front face of the obstacle. Error bars reflect 95% confidence intervals with between-subjects variability removed. (A) By obstacle height and viewing window. (B) By obstacle depth and viewing window.

**Lead foot elevation and clearance** As shown in Fig 12, subjects elevated the lead foot to a greater height when the obstacle was taller (*χ*^2^(1) = 4.60, p<.05, dAIC = -2.60) and deeper (*χ*^2^(1) = 6.34, p<.05, dAIC = -4.34). The tendency for subjects to increase the maximum foot elevation for taller and deeper obstacles was also found in Experiment 1. However, foot elevation in Experiment 2 was consistently greater than it was in Experiment 1 even in the full vision condition, despite the fact that trials in the full vision condition were identical in both experiments. This clearly reflects the adoption of a more cautious strategy due to the increased difficulty of the task in Experiment 2.

Adding viewing window as a predictor significantly improved the model of lead foot elevation relative to the height-only model (*χ*^2^(4) = 33.46, p<.001, dAIC = -25.46). Further adding height × viewing window resulted in an increase in AIC, suggesting that the effect of obstacle height did not significantly vary across VW conditions. Contrasts with Dunnett’s correction between each VW condition and the full vision condition were significant (p<.05) only in the VW-1 condition. Thus, subjects’ ability to elevate their lead foot over short and tall obstacles was affected only when the obstacle did not become visible until the last step. This was also the case in Experiment 1. However, whereas lead foot elevation in VW-1 was greater than or comparable to that in full vision in Experiment 1 (see Fig 5, it was significantly lower compared to full vision in Experiment 2. This is likely a consequence of having insufficient time to properly position the trail foot before the obstacle. By having to place their trail foot closer to the obstacle than normal, subjects had less time to elevate the lead foot to the same height as in full vision (see [2] for a similar effect.)

**Fig 13:**
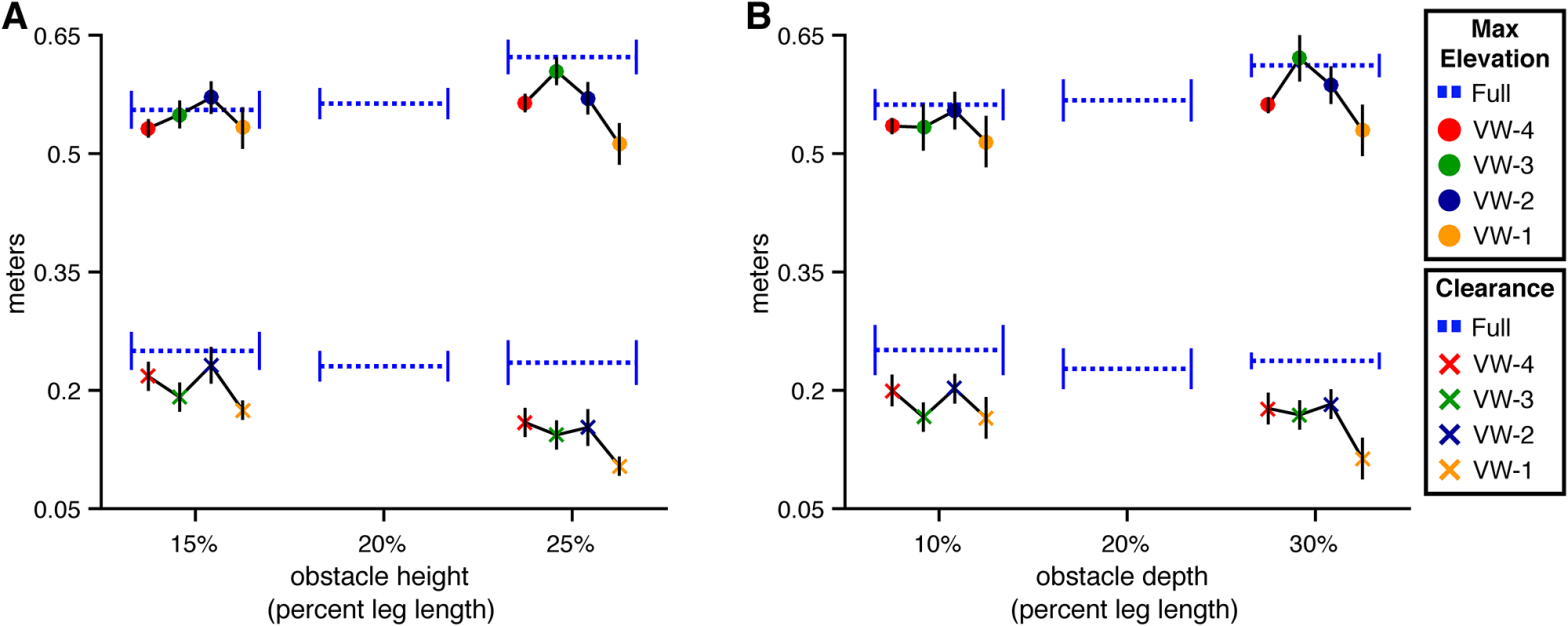
Maximum height and clearance of the trailing foot for Experiment 2. Maximum step height (top) and obstacle clearance (bottom) of the toe marker placed on the foot at the time it crossed over the front face of the obstacle. Error bars reflect 95% confidence intervals with between-subjects variability removed. (A) By obstacle height and viewing window. (B) By obstacle depth and viewing window.

**Trail foot elevation and clearance** Trail foot elevation was significantly affected by both obstacle height and viewing window (*χ*^2^(5) = 25.93, p<.001, dAIC = -15.93 compared to the baseline model) but the interaction between these two variables was not statistically significant (*χ*^2^(4) = 8.09, p = .08, dAIC = -0.09) (see Fig 13A). Contrasts with Dunnett’s correction between each VW condition and the full vision condition revealed significant differences (p<.05) in the VW-4 and VW-1 conditions.

The analysis of the effects of depth and viewing window on trail foot elevation revealed a similar pattern of results (see Fig 13B); that is, trail foot elevation was significantly affected by both obstacle depth and viewing window (*χ*^2^(5) = 28.38, p<.001, dAIC = -18.38 compared to the baseline model) but the interaction between depth and viewing window was not statistically significant (*χ*^2^(4) = 5.26, p = .26, dAIC = 2.74).

Taken together, this analysis suggests that walkers are able to properly elevate their trail foot over obstacles of varying heights and depths as long as visual information about the obstacle was available between roughly two and three step lengths in advance. If such information is available before or after this portion of the approach phase, the walker’s ability to properly elevate the trail foot will be degraded. Furthermore, because these effects were observed in Experiment 2 but not in Experiment 1, we can conclude that the degradation is due to the absence of information about obstacle location specifically.

#### Collision rates

**Fig 14:**
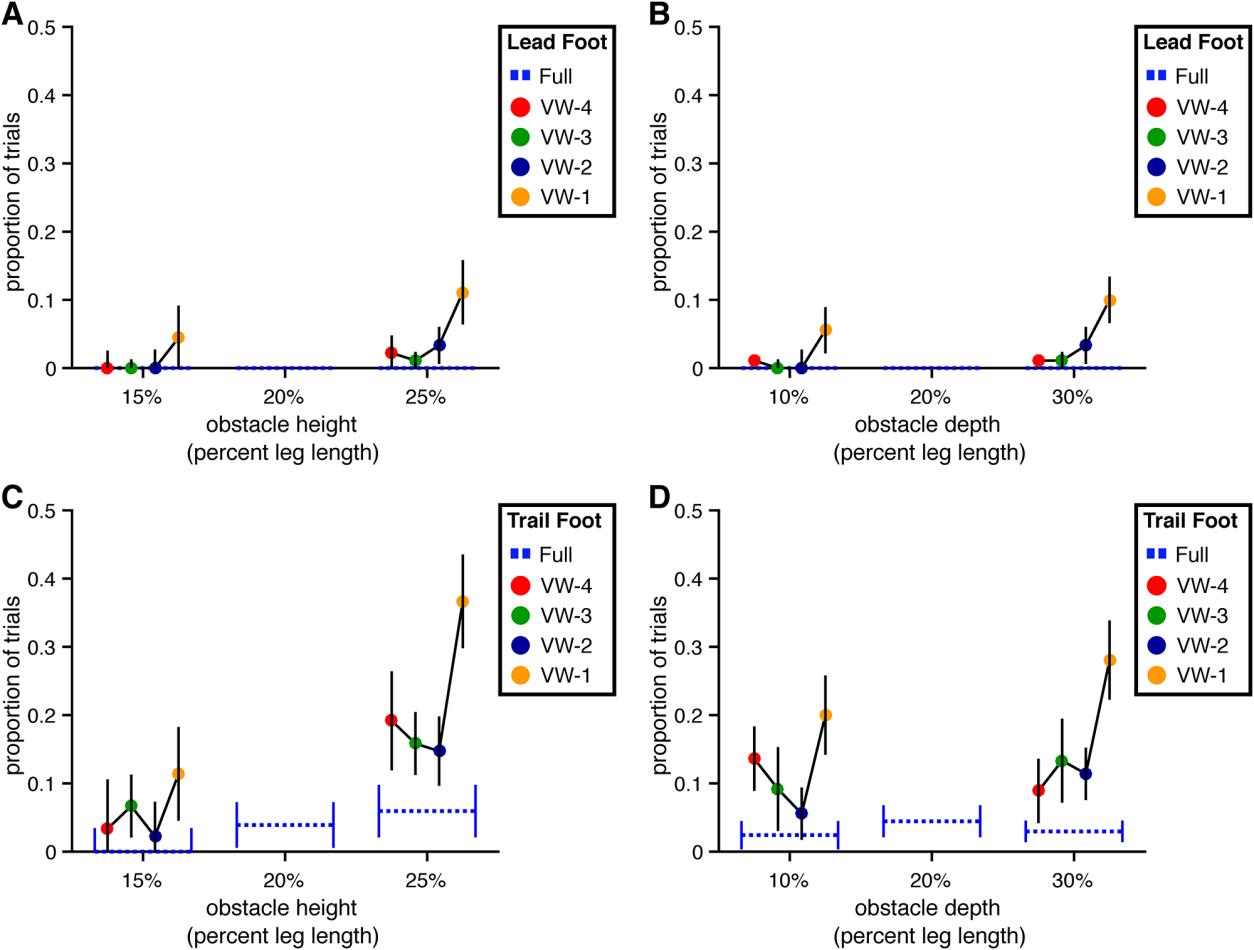
Collision rates for Experiment 2. The percentage of trials with a collision between the obstacle and the lead and trailing foot are presented by obstacle height and depth. Error bars reflect 95% confidence intervals with between-subjects variability removed. (A) Collisions with the lead foot by obstacle height. (B) Collisions with the lead foot by obstacle depth. (C) Collisions with the trailing foot by obstacle height. (D) Collisions with the trailing foot by obstacle depth.

As in the full vision condition of Experiment 1, collisions were rare for the lead foot but more frequent for the trail foot (see Fig 14). Lead foot collision rate was not significantly affected by either height (*χ*^2^(1) = 3.51, p = .06, dAIC = -1.51) or depth (*χ*^2^(1) = 2.39, p = .12, dAIC = -0.39), but the addition of viewing window as a predictor did improve the model (*χ*^2^(4) = 11.93, p<.05, dAIC = -3.93). This effect is likely to be a consequence of the reduced foot placement distance before the obstacle in the VW-1 condition.

Trail foot collision rate was significantly greater for taller obstacles (*χ*^2^(1) = 5.49, p<.05, dAIC = -3.49) but was not affected by the manipulation of obstacle depth (*χ*^2^(1) = 1.55, p = .21, dAIC = 0.45). The manipulation of viewing window also affected trail foot collision rate (*χ*^2^(4) = 15.64, p<.01, dAIC = -7.64), in particular in the VW-1 and (to a lesser degree) the VW-4 conditions.

Overall, the rate of trail foot collisions in the full vision condition was lower in Experiment 2. This may initially seem surprising given the greater difficulty of the task in Experiment 2 but can be readily understood as a consequence of the cautious strategy that subjects adopted in Experiment 2 when obstacle location information was not continuously available.

#### Summary

The results of Experiment 2 differed from those of Experiment 1 in two main respects. First, subjects behaved more cautiously in Experiment 2 compared to Experiment 1. They tended to walk slower during the approach phase and elevate their feet to a greater height during obstacle crossing. These differences were observed even in the full vision condition of Experiment 2, which was identical to that of Experiment 1. Second, the effects of the viewing window manipulation were stronger and observed in more viewing window conditions (VW-4 and VW-1) in Experiment 2 compared to Experiment 1. Taken together, the results indicate that obstacle crossing performance degrades when information about obstacle location is not available when it is needed to guide placement of the feet in front of the obstacle. This contrasts with the information about obstacle size, which can be sampled at any point during the last few steps before placement of the lead foot before the obstacle, as demonstrated in Experiment 1. Thus, walkers have less flexibility in when they can sample information about obstacle location compared to information about obstacle size.

## General discussion

### Summary of main findings

To successfully cross an obstacle in the path of locomotion, humans must adapt their gait to both the location of the obstacle and its dimensions (e.g., height, depth). Walkers are most successful in obstacle crossing when they can sample information about location shortly before placement of the trail foot in front of the obstacle, which makes sense given that precise foot placement plays a key role in minimizing the risk of tripping [2, 3, 16]. The results of the present study suggest that the success of obstacle crossing is less dependent on when information about obstacle size is sampled. That is, walkers can accommodate variations in obstacle height and depth when information about these properties is detected several steps in advance or much later when such information is detected as late as the last step before obstacle crossing.

The ability to use information about obstacle size that was detected several steps in advance was demonstrated in Experiment 1, in which behavior in the VW-4 condition was similar to that observed in the full vision condition on measures of number of steps, walking speed, lead and trail foot placement, maximum foot elevation, foot clearance, and number of collisions. On measures where behavior was affected by manipulations of obstacle height and depth in the full vision condition, behavior was similarly affected in the VW-4 condition. In both conditions, subjects placed their feet slightly closer to the obstacle when it was deeper but not when it was taller, and elevated their feet to a greater height when the obstacle was taller and when it was deeper. Although behavior in the VW-4 condition was also similar to the full vision condition in Experiment 2, when the line marking obstacle location was absent, performance in both conditions was degraded compared to the corresponding conditions in Experiment 1. Thus, the ability to use information that was sampled in advance is specific to information about obstacle size.

Why are walkers able to use information about obstacle size that is sampled in advance but need information about obstacle location later during the approach? One possibility is that size and position (relative to the walker) differ in their stability. Information about properties that do not vary from moment to moment, such as obstacle size, may be sampled well in advance because their stability makes it possible for that information to be useful at a later time. In contrast, information about properties that vary from moment to moment, such as the walker’s position relative to the obstacle, must be sampled around the time that it is needed for making gait adjustments. A similar account has been proposed to explain why the transport and manipulation components of reaching and grasping are affected differently when there is a delay between viewing the object to be picked up and initiating movement of the hand (see [32]). Of course, this is only true when the dimensions of the obstacle (or object to be picked up, in the case of reaching and grasping) are actually stable. If the obstacle is a pet that may decide to stand up just as the walker is stepping over it, information about height and depth that is sampled in advance would be much less reliable.

The ability to use information about obstacle size later in the approach was apparent in the similarity of behavior in the VW-2 and full vision conditions of Experiment 1. Although the obstacle did not appear until shortly before placement of the lead foot, subjects generally elevated their feet to a greater height for taller and deeper obstacles as they did in the full vision condition. Nevertheless, some of these effects were weaker than they were in the full vision condition, suggesting that subjects’ ability to adapt to changes in obstacle dimensions was slightly diminished in this condition. When the obstacle did not appear until the last step before crossing (VW-1 condition), subjects were even less able to tailor foot elevation to variations in obstacle height and depth. Thus, the last point at which subjects are able to adapt gait to obstacle size appears to be shortly before placement of the trail foot in front of the obstacle.

Why are walkers able to adapt to changes in obstacle size even when the relevant information is not made available until shortly before obstacle crossing? Even when information about obstacle size is available throughout the approach, walkers apparently do not make any adaptations to gait that are specific to obstacle size prior to the step over the obstacle. (The one exception is the small change in foot placement in front of the obstacle as a function of obstacle depth.) As such, not knowing the dimensions of the obstacle prior to the initiation of obstacle crossing does not impair performance in the way that not knowing the location of the obstacle does.

### Broader significance

#### Functional significance

The findings of the present study demonstrate that walkers are capable of adapting gait to variations in obstacle height and depth even if information about these characteristics is sampled several steps in advance. One might wonder about the significance of this ability during real-world obstacle crossing given that walkers must sample information about obstacle location later during the approach anyway (to guide foot placement in front of the obstacle), and could sample information about obstacle dimensions at the same time. In other words, what is the functional significance of estimates of obstacle dimension based on information that is detected well in advance if those estimates are updated later during the approach, when information about obstacle location must be sampled?

Although walkers need information about obstacle location when they are closer to the obstacle, they do not necessarily need to look at the obstacle to pick up that information. In many complex environments, there are other objects that are visible in the upper visual field that could be used to keep track of one’s position relative to the obstacle without actually looking at it. For example, if the obstacle is a fallen branch that lies on a hiking path near the base of a tree, the walker could perceive his or her changing position relative to the branch without looking down or relying on peripheral vision by detecting information about the relative position of the tree, much like subjects in the study by Rietdyk and Rhea [12] relied on vertical posts when the lower visual field was occluded. Although the tree provides a reference for tracking one’s position relative to the obstacle without looking down, it does not provide any information about obstacle size. However, because walkers are able to sample information about obstacle size and use that information later to guide the trajectory of the feet over the obstacle, they can negotiate the obstacle by relying on the reference object (e.g., the nearby tree) to track obstacle location and memory to adapt gait to obstacle dimensions.

This could be especially useful when there are multiple obstacles in the path of locomotion. In such situations, walkers must shift their gaze to future obstacles before stepping over the obstacle that is most immediately in front of them [6]. The ability to use information about obstacle dimensions that is sampled in advance to guide obstacle crossing at a later time may play a key role in negotiating such terrain. Future studies should investigate the robustness of this ability in the context of multiple obstacles.

#### Methodological significance

The present study is one of a small number on obstacle crossing to be conducted in a virtual environment (VE) viewed through a head-mounted display (HMD). VEs offer a potentially powerful tool for investigations of visually guided locomotion over complex terrain, as they enable researchers to manipulate visual information in a manner that is not possible in the real world. Of course, there are also some drawbacks. Misperceptions of size and egocentric distance (which are well documented in VEs; e.g., [33–35]), latency in updating images of the VE within the HMD following head movement, the relatively small field-of-view of the HMD (76°H x 64°V per eye), and minor imprecisions in the alignment of the real and virtual feet could lead to behavior that differs from that in the real wold. This could explain why subjects in the present study behaved more cautiously when stepping over the obstacle. Whereas lead foot clearance in previous studies of obstacle crossing conducted in the real world was 12 to 20 cm [2, 4, 5, 13–15, 18, 36], subjects in Experiment 1 (full vision condition) of the present study cleared the obstacle by 20 to 30 cm on average. The use of VE could also account for the elevated trail foot collision rate, which ranged from 20 to 30% in Experiment 1.

Despite these differences, subjects were consistent in the placement of their lead and trail feet in front of the obstacle, elevated their feet to a greater height for taller and deeper obstacles, and avoided making obstacle contact with the lead foot on the vast majority of trials. Thus, in many respects, obstacle crossing behavior in the present study was similar to that observed in the real world. Given the potential for using VEs in both basic research on locomotion as well as in the diagnosis and treatment of disorders that affect mobility (e.g., gait training following stroke), further research on obstacle crossing behavior in virtual environments, as well as comparisons of behavior in real and virtual environments, would make a valuable contribution.

### Conclusions

Although walkers need information about both the location and size of an upcoming obstacle to guide successful crossing, only information about obstacle location is needed at a specific point in time during approach. The success with which walkers are able to negotiate obstacles is less dependent upon when information about obstacle height and depth is sampled. They can sample information about obstacle size several steps in advance and use that information to adapt the placement and trajectory of the feet during obstacle crossing. Alternatively, they can sample such information as late as the last step before obstacle crossing and still elevate their feet to different heights depending on the dimensions of the obstacle.
